# Health Professional Perceptions of Delivering Hospital Falls Prevention Education—A Qualitative Study

**DOI:** 10.1177/01939459251319078

**Published:** 2025-02-13

**Authors:** Anne-Marie Hill, Cheng Yen Loo, Steffanie Coulter, Carol Watson, Sharmila Vaz, Meg E. Morris, Leon Flicker, Tammy Weselman

**Affiliations:** 1 School of Allied Health, University of Western Australia, Perth, WA, Australia; 2 WA Centre for Health and Ageing, University of Western Australia, Perth, WA, Australia; 3 School of Arts and Humanities, Edith Cowan University, Perth, WA, Australia; 4 Royal Perth Bentley Group, East Metropolitan Health Service, Perth, WA, Australia; 5 Ngangk Tira Institute for Change, Murdoch University, Perth, WA, Australia; 6 Academic and Research Collaborative in Health, and Care Economy Research Institute, La Trobe University, Melbourne, VIC, Australia; 7 Victorian Rehabilitation Center, Glen Waverley, Melbourne, VIC, Australia; 8 Geriatric Medicine, Medical School, University of Western Australia, Perth, WA, Australia

**Keywords:** accidental falls, falls prevention, hospitals, behavior change, patient education

## Abstract

**Background:**

Providing patient falls prevention education can help reduce falls in hospitals, yet research exploring staff perceptions about providing falls education in hospitals is limited.

**Objective:**

We sought to determine enablers and barriers to implementing a hospital falls prevention education program (the Safe Recovery Program) from the clinical staff perspective.

**Methods:**

Purposive sampling was used to recruit health professionals (N = 40) from 12 acute medical and surgical wards at a 450-bed hospital in Perth, Western Australia. Participants were given the option to take part in a focus group or semi-structured interview. Data were analyzed via directed content analysis.

**Results:**

Findings were distinguished into 2 themes, being the barriers and enablers to implementing the Safe Recovery Program. Enabler subthemes were the mode and medium of delivering the program, the use of repetition to instill the learnings, identifying who is best to deliver the program, and utilizing the role of informal carers to reinforce the education. Barrier subthemes were patient cognitive impairments and patient illness, patient risk-taking behavior, timing of program delivery according to patient readiness, time and resource shortage, and communication barriers with non-English speaking patients.

**Conclusion:**

A comprehensive approach to program delivery can enable health professionals to implement evidence-based falls prevention education in hospitals. Extant factors must be considered during the implementation phase to ensure the Safe Recovery Program is sustainable and to optimize patient uptake of falls prevention education.

Falls in hospitals are a serious and widespread patient risk and remain one of the most frequently reported patient safety incidents, with 30% to 50% resulting in injuries.^1-4^ An injurious fall can result in fractures, traumatic brain injuries, death,^2,3,5,6^ and psychological distress.^7^ Older hospital patients are particularly vulnerable to falls,^2,3,8,9^ and those who fall^2,3,8,9^ are more likely to experience subsequent falls, extended length of stay, and readmissions.^6,9^

A rapid review that recently synthesized evidence for falls prevention at hospitals noted that health professionals sometimes alter their care practices by restricting patient movement and ambulation to reduce falls risk.^5^ This practice is recognized to have poor patient outcomes and can contribute to patient functional decline.^10^ Systematic reviews suggested a multifactorial falls prevention approach can reduce falls in hospitals.^2,3,8,11,12^ Nurturing a safety culture at hospitals coupled with greater patient knowledge of falls risks can promote positive behavioral changes to reduce risk-taking and thereby reduce falls.^3,11,13^

This emphasis on embedding falls prevention education into unit culture and care procedures in hospitals is supported by the World Falls Guidelines (Grade 1A evidence).^4,6,14^

Promoting positive behavioral change is underpinned by the Capability-Opportunity-Motivation Behavior (COM-B) model, a framework used for characterizing and designing behavior change interventions.^15^ According to the COM-B model, capability is defined as having the necessary knowledge and skills to engage in a specific activity, such as preventing falls in a hospital.^16^ Motivation is defined as the brain processes that energize and direct behavior such as habitual processes, emotional responses, and decision making. Opportunity is defined as the factors that lie outside the individual that make the behavioral change possible.^15,16^

The Safe Recovery Program is an Australian hospital patient falls prevention education program that uses individualized education and goal setting as its intervention mechanism, common among behavioral change interventions.^17,18^ The program is provided to hospitalized older patients or patients deemed at significant risk of falling with good cognition or mild cognitive impairment (it is not directly provided to patients with moderate or severe cognitive impairment). Validated in 3 randomized controlled trials, implementation of the program at the ward level demonstrated falls-related reduction by 35%.^17-19^

The Safe Recovery Program has proven to be an effective intervention for reducing patient fall rates in hospitals and promotes positive behavioral change on a ward and individual (patient/staff) level. Studies evaluating the Safe Recovery Program have identified that the program’s mechanism of effect is that it helps to create a positive work culture around falls prevention in hospitals and increases staff knowledge and awareness of maintaining a safe ward environment.^20,21^ Similarly, from a patient perspective, those who received the program have reported that identifying falls prevention strategies and developing behavioral goals around addressing fall risks helped raise their awareness, knowledge, and confidence on how to remain safe in the hospital.^6^

The program is currently delivered by trained allied health professionals who seek to raise patient and staff awareness and knowledge of falls and their prevention in hospitals, facilitate goal setting of falls risk reduction strategies, and empower the patient to enact their strategies.^14^ Nevertheless, qualitative studies indicated that implementing patient education is challenging.^22,23^

While revisions to the Safe Recovery Program have been completed to facilitate implementation in modern hospital settings, many implementation initiatives fail because of an absence of adaptive tailoring for context-specific implementation, which requires effective stakeholder consultation and planning central to implementation science theory.^24^ Effective implementation requires consultation with key stakeholder groups to determine barriers and enablers. The objective of this study was to determine enablers and barriers to implementing the Safe Recovery Program from the clinical staff perspective.

## Methods

### Design

Semi-structured focus groups and one-on-one interviews were conducted with nurses, physiotherapists, occupational therapists, and a physician in a quiet and private conference room at 1 large Western Australian (WA) tertiary hospital to capture a summative appreciation of staff perceptions of the implementation of the Safe Recovery Program.^25^ Participants unable to attend an in-person interview were given the option to participate on the telephone and virtually via a secure online platform.

Guided by the interacting components that inform behavioral change, capability, opportunity, and motivation, a discussion guide for the facilitator was developed to probe the barriers and enablers to implementing the Safe Recovery Program (Table 1).^15^ A total of 20 interviews and 2 focus group sessions (n = 10 participants each) were conducted. The focus group and interview guide were designed to elicit conversation and in-depth discussions among the participants. Focus groups aided the researchers to identify consensus, diversity of opinions, and experiences among the participants. The interviews enabled the researchers to gain an emic perspective on social phenomena.^26,27^

**Table 1.** Focus Group and Interviews Discussion Guide for Facilitator.

Steps involved in the focus group and interview	
Discussion 1	Discuss your experiences with fall prevention education in hospitals
Discussion 2	Exploring how clinicians identify patients at risk of falls and strategies to direct fall prevention education to these patients
Discussion 3	Viewing the Safe Recovery Program’s (SRP) resources and reflecting on the material (video + workbook) Participants will be shown a newly revised patient-tailored, evidence-based fall prevention education resource called the Safe Recovery Program Participants to share their views about the resource and the barriers and enablers to using it in WA hospitals, including how they think older people with and without common problems identified in Step 2 (cognitive impairment, medically unwell) would respond/enact messages
Discussion 4	Brainstorm how and where fall prevention messages might be delivered to older people during or before hospital admission and ways to support nurses and health professionals in delivering fall prevention education Commonly based barriers and enablers identified in previous systematic reviews and realist evaluations of SRP to be used to probe
Closing discussion	Group member checking and closing the focus group
Interview question guide for focus group and 1:1 interview	
Step 1: Your experiences with fall prevention education in hospitals	
Step 2: Exploring how clinicians identify patients at risk of falls and strategies to direct fall prevention education to these patients	
(a) How do you identify patients at risk of falls?	
(b) How could fall prevention education messages be directed to patients at risk of falls?	
(c) Who do you think should be responsible for delivering fall prevention education to hospital patients?	
Step 3: Viewing the Safe Recovery Program’s resources and reflecting on the material (video + workbook)	
Step 4: Brainstorm how and where fall prevention messages might be delivered to older people during or before hospital admission and ways to support clinical staff (nurses, allied health professionals, and physicians) in delivering fall prevention education	
(a) Patient-level barriers and enablers	
(b) Intervention-staff level barriers and enablers	
(c) Intervention-level barriers and enablers	
(d) System level barriers	

### Ethical Considerations

Ethical approval was received from the Human Research Ethics Committees of Royal Perth Hospital (GRS No: 5775). All participants were provided with a plain language information sheet and given a verbal explanation of the aims of the study and what was required of them. Participants were given the option to attend a focus group or interview and were informed that their participation was voluntary. Participants were informed that all information collected would be confidential and de-identified to protect their privacy. Their right to withdraw and discontinue were communicated verbally and in writing. Each participant provided written informed consent before commencing the study.

### Setting, Recruitment, and Sampling

Clinical staff were recruited from 12 acute medical and surgical wards at a 450-bed hospital in WA. Participants were recruited via purposive sampling—a process of selecting respondents to yield appropriate and useful information to increase the depth of understanding of a subject matter.^28^ The research team included a hospital employee (SC) who distributed the recruitment material in the medical and surgical wards and engaged with the Allied Health and Nursing Unit manager to identify suitable candidates to invite to take part in the focus groups and interviews.

### Data Collection

The research team were experienced health professionals with research training (AMH, MM, CYL, SV, TW, and LF) and hospital allied health staff providing research-focused support (SC and CW). The research team has extensive experience working directly with older people on research projects in the community and hospital settings. Focus groups and interviews were conducted at a convenient time and location for the participants by researchers AMH, SC, or TW. The focus group discussions and interviews used the same guided questions. All focus groups and interviews were audio-recorded and transcribed verbatim using an online transcription service.

### Data Analysis

Interview and focus group data were coded and analyzed using directed content analysis, a commonly used data analysis method in healthcare research.^29,30^ The transcripts were reviewed by 3 members of the research team (AMH, CYL, SC) to become immersed in the data. Guided by the research question, textual data were highlighted and indexed into general codes as a process of making sense of the textual data.^31^ Codes refer to labels that assign symbolic meaning to the descriptive information compiled during the study to make sense of the data.^32,33^ The codes were then grouped into units of meaning and sorted into categories called themes.

### Rigor and Trustworthiness

Three research team members (AMH, CYL, SC) independently reviewed the transcripts alongside the original recordings to ensure accuracy. The 3 researchers then discussed the relationships between the different groups of codes to form the overarching themes. This method enabled investigative triangulation—a process of making sense of data by corroborating and refuting interpretations to achieve intercoder data agreement.^34,35^ Member checking was important to establish the credibility and validity of the findings.^36^ As such, the codes and themes were summarized and presented to the research participants to check for accuracy and to allow for further clarification of the participants’ experiences, as required.

## Results

A total of 40 participants were involved in the study, consisting of physiotherapists (n = 18, 45%), occupational therapists (n = 8, 20%), nurses (n = 13, 32.5%) and 1 physician (n = 1, 2.5%). An overview of the participants’ demographic data and recruitment methods by profession can be found in Table 2. Two focus group sessions were hosted at a convenient location within the hospital where 10 participants attended each session. Participants were given the option to attend a focus group, one-on-one interview, or both to accommodate their busy schedules during working hours (Table 3). One clinical nurse participated in a focus group and individual interview. A further 19 participants took part in individual interviews but not a focus group. Most of the health professionals were engaged in full-time employment (n = 32, 80%) providing direct patient care (n = 39, 97.5%) either in medical, surgical, geriatric, or emergency wards.

**Table 2.** Health Professional Participant Demographics (N = 40).

Participant characteristics	n (%)
Age range	
20-34	27 (67.5%)
35-44	6 (15%)
45-54	3 (7.5%)
65+	2 (5%)
Prefer not to say	1 (2.5%)
Gender	
Female	37 (92.5%)
Male	3 (7.5%)
Profession	
Physiotherapist	18 (45%)
Occupational therapist	8 (20%)
Nurse	13 (32.5%)
Physician	1 (2.5%)
Education level	
Bachelor’s degree	30 (75%)
Graduate certificate/diploma	3 (7.5%)
Post-graduate qualification	6 (15%)
Other	1 (2.5%)
Primary language	
English	37 (92.5%)
Other	3 (7.5%)
Employment status	
Full-time	32 (80%)
Part-time	8 (20%)
Primary role	
Direct patient care	39 (97.5%)
Management	1 (2.5%)
Work area	
Medical	22 (55%)
Surgical	4 (10%)
Gerontology	7 (17.5%)
Emergency	7 (17.5%)
Years in profession	
0-2 years	3 (7.5%)
3-5 years	13 (32.5%)
6-10 years	11 (27.5%)
>11 years	13 (32.5%)
Received falls education in last 12 months	
Yes	14 (35%)
No	26 (65%)

**Table 3.** Data Collection Method and Staff Recruitment (N = 40).

Data collection method	Profession	Number of participants
Interview	PT (n = 14), OT (n = 2), CN (n = 1), PHY (n = 1)	Participants (n = 19)
Focus group 1 (12/07/2023)	EN (n = 1), RN (n = 7), SDN (n = 1), NUM (n = 1)	Participants (n = 10)
Focus group 2 (13/07/2023)	OT (n = 6), PT (n = 4)	Participants (n = 10)
Interview and focus group 1	CN (n = 1)	Participant (n = 1)

Abbreviations: PT: physiotherapists; OT: occupational therapists; CN: clinical nurse; PHY: physician; RN: registered nurse; SDN: staff development nurse; EN: enrolled nurse; NUM: nurse unit manager.

### Theme 1: Enablers to Patient Update of Education Program

#### Subtheme 1.1: Mode and Medium of Program Delivery

To improve the likelihood of patient engagement with the Safe Recovery Program, participants said having different modes of communication was important. One highlighted strength was being able to offer a video, booklet, or both, to allow patients to choose how they engage with the education: “If you have got a patient that’s got a cognitive or hearing impairment, you’ve got different mediums to give the information” [P2].

The use of actors in the video to simulate fall risks was also recognized as a helpful way to increase patient engagement, however, staff recognized that age is not the only determinant of fall risk in the hospital setting. An example described by P36 was younger patients (<65 years) with acute neurological conditions affecting their mobility. The casting of both younger and older-looking actors was noted as a way that could ensure patients from different age groups could relate to the training: “I think as a younger person watching this video, I’d be like, oh well, that’s just for older people” [P36].

#### Subtheme 1.2: Repetition of Messaging and Learnings

Many participants reported that repetitive, yet simple program messaging effectively heightened patient engagement. According to P34, the Safe Recovery Program^37^ “empowers them [patients] to think of their own goals and their own strategies.” Participants perceived that the design of the program encourages patients to take greater responsibility for their own safety and also moved from a reactive model of care, toward a preventative approach.^38^

#### Subtheme 1.3: The Most Suitable Health Professionals to Deliver the Safe Recovery Program

Physiotherapists and occupational therapists were identified as the most suitable health professionals to deliver the Safe Recovery Program because of their frequent contact with patients and training. According to P1, “I feel like physios are really well placed to provide that education because of their background from a mobility perspective.”

However, there was the acknowledgment that the Safe Recovery Program could be delivered by any allied health professional if given guidance from senior clinicians: “Definitely could be physio assistant if you keep it nice and simple. . . We could teach’ em kind of the words they need to use” [P2].

#### Subtheme 1.4: Informal Caregivers to Reinforce Safe Recovery Program Training

Some participants recognized family and other informal careers as an important resource^39-41^: “I think they’re [family/friends] a good reinforcer. They’re not going to give the initial information but getting them to assist is reasonable” [P4]. At a minimum, providing falls prevention information to informal caregivers was considered important: “Perhaps the booklet could be given when family members are around so the patient can watch the video and a family member can reinforce from the booklet” [P6].

### Theme 2: Perceived Barriers to Patient Uptake of Education Program

Overall, participants recognized the benefits of implementing falls prevention education to hospital patients. However, concerns were sometimes raised about the difficulty of implementing the Safe Recovery Program in some acute hospital wards. According to P29, “Often our patients have such a short stay and a quick turnaround, they’re often inundated by people.” As the Program utilizes a booklet, video, and verbal education, some participants questioned whether it contained too much information to process: “It’s quite a long video. It’s harder to get people to concentrate when there’s so much going on or people coming in and out” [P1]. Some participants suggested the Safe Recovery Program was better suited for the longer-stay wards where there are less disruptions to patients’ time: “It might be hard to engage in a 9-minute video and write goals when you’re unwell. This program would be so good in a slower stream setting” [P33]. However, some participants felt that delivering the Safe Recovery Program in the acute hospital setting was essential because many inpatient falls occurred within the first 24 hours after admission: “Falls education is usually low on the list of priorities, but it’s extremely important particularly when patients are at their most vulnerable” [P4].

#### Subtheme 2.1: Cognitive Impairment and Illness of Patients

Some participants raised concerns about the ability of patients with impaired cognition to engage with the Safe Recovery Program. Cognitive impairment attributed to acute illness or a pre-existing condition led some participants to question if more simplified versions of the training should be available: “I think cognitive impairments will be a huge challenge. I’m not sure how they would engage with the education” [P2].

#### Subtheme 2.2: Risk-Taking Behavior Among Some Patients

Patients who are less receptive to receiving education and more prone to risk-taking may also present a barrier to successful Safe Recovery Program implementation: “We’ve got a lot of impulsive patients that just do their own thing regardless of the advice we give them” [P2].

Participants stated that delay in responding to calls for assistance could trigger patients to take risks to ambulate unassisted to the toilet: “They will often say ‘I’m ringing the bell, no one’s coming.’ Unfortunately, the reality on our wards is that people aren’t getting answered that quickly” [P31]. Staff perceived that minimizing risk-taking behavior would require improvements to the rate ward staff can respond to requests for assistance. According to P18, “the wait time before ward staff respond can be up to 30 minutes.”

#### Subtheme 2.3: Timing of Program Delivery According to Patient Readiness

The timing of when to deliver the Safe Recovery Program was raised by several participants. The readiness of patients to engage with the education was deemed important. Implementing the program when a patient is first admitted could be problematic if the patient was medically unwell or having difficulty coping with their health condition: “I would say it’d just be picking the timing because if you gave it pre-op, they might be delirious, and at post-op, they might not retain it” [P7].

#### Subtheme 2.4: Time and Resource Shortage to Deliver the Safe Recovery Program

While most participants felt it was important to draw upon the clinical expertise of physiotherapists and occupational therapists to initiate the program, time constraints and staff resources remained a concern. According to P31, “I don’t know if there would be enough time for therapists to implement it. It could potentially get implemented in a shorter version.”

Some participants suggested appointing a dedicated “champion”^42^ to actively promote the program through critical stages of implementation and hold staff accountable. This sentiment was reflected in P39’s statement: “Having someone dedicated to setting it up and then having someone to champion it would be ideal.” The participants explained that the champion would need to be someone who “*valued* the importance of reducing falls risk, had a strong background in falls prevention, and enjoyed talking to patients” [P31].

#### Subtheme 2.5: Communication Barriers With Non-English Speaking Patients

Lastly, the accessibility of the Safe Recovery Program to people with limited English language skills was raised as a barrier. The State of Western Australia has a large culturally and linguistically diverse population, where over 32.2% were born overseas, and the proportion who speak a language other than English has grown from 14.5% in 2011 to 17.7% in 2016.^39,40^ At present the Safe Recovery Program is only available in English which indirectly excludes non-English speaking patients from engaging with the program without the assistance of an interpreter or family member. This led several participants to query: “Are we going to put it [the Safe Recover Program] in a different language?” [P3]

## Discussion

Health professionals in this study agreed that providing falls prevention education to hospital patients is important and should be prioritized. This finding is supported by previous studies conducted with hospital health professionals.^21,43^ In particular, the Safe Recovery Program was deemed unique because it empowered patients to engage in active learning to improve their knowledge about falls risks during their admission.

Most participants perceived the Safe Recovery Program could be delivered by nursing, physiotherapy, occupational therapy, or therapy assistant staff with the provision of adequate supervision. Physiotherapists and occupational therapists were often deemed the most suitable, and appointing a champion to promote the Safe Recovery Program was suggested to help embed the program into usual care and engender a positive cultural shift.

Previous trials of the Safe Recovery Program were delivered by a physiotherapist and this was found to help reduce falls through the process of building the patients’ understanding of how to initiate safe behaviors within the hospital setting.^14,19^ A recent randomized controlled trial also revealed that providing staff training, ample time and resources helped to support the delivery of falls prevention education, and raise patient motivation and understanding of how to reduce falls and injuries.^3^ In this study, many health professionals also highlighted the importance of receiving training and having access to sufficient time and resources to successfully implement the Safe Recovery Program.

Delivering information in a multimedia format enabled patients to engage in their preferred mode of learning consistent with adult learning principles^37^ while the repetition of instructions was a helpful technique to reinforce the messaging for older adults.^21^ Many health professionals in this study also emphasized the importance of enabling patients to access education in a variety of ways and to repeat the learnings to transform it into practice.

The Safe Recovery Program was perceived as a tool to empower patients to manage their safety. This concurred with findings from the original and revised testing that patients and staff both found the program useful for making shared safety goals.^6,14^ This ethos of delivering education with a focus on “preventative care” was noted as a divergence from the reactive approach to care in hospitals.^43^

Some participants perceived that effective implementation could be difficult in the acute hospital setting due to patient factors and resource limitations. Recent studies^21,44^ also identified staff time and workload demands as major barriers to the implementation of evidence-based practices.

Capitalizing on informal careers to reinforce the training was suggested when patients present with cognitive impairments or lack the English language skills to comprehend the information without a translator. This concurs with evidence that the Safe Recovery Program is delivered to adults with no to mild cognitive impairment.^14,18^ For patients with dementia or delirium, careers and staff are recommended to provide assistance for safe mobility.^21^

Composite versions of the Safe Recovery Program in different languages could be developed in future iterations of the program to broaden the accessibility of the information to culturally diverse hospital patients. This is particularly important because Western Australia has a large culturally and linguistically diverse population, where over 32.2% were born overseas, and the proportion who speak a language other than English has grown from 14.5% in 2011 to 17.7% in 2016.^39,40^

Staff also noted patient risk-taking would be good to address as recent reviews have found that education interventions can reduce risk-taking attributed to patients’ personality, desire to remain independent, embarrassment associated with seeking assistance and never having fallen in hospital.^45,46^ Identifying the best time to implement the program was viewed as a key enabler, as multiple factors, including patients’ medical stability, can contribute to the patient’s ability to focus and engage. This finding is consistent with another study that found patient readiness to engage with the Safe Recovery Program to be an important factor to consider.^43^ Further research should focus on ways to tailor falls prevention education to individual patient needs during their hospital admission.

### Strengths and Limitations

Participants were recruited from 1 WA public hospital, which limits generalizability. While purposive sampling ensured health professionals with different clinical expertise contributed, each hospital ward had a distinctive work culture that could have influenced participant responses.^20,44^ We did not capture opinions on whether the Safe Recovery Program improved patient outcomes, despite the focus on patient-centered care.^45^ Strengths of the study include the recruitment of participants via purposeful sampling to garner expert opinions. The interviews were semi-structured to facilitate in-depth yet targeted discussions.^46^ We used directed content analysis techniques to analyze the data which is a well-established data analysis method in health science research. Data triangulation and member checking throughout the study established rigor and ensured visibility, comprehensibility, and transparency of the findings.^33,47^

## Conclusion

Hospital multidisciplinary staff recommended embracing a behavioral change approach to falls prevention education in ward settings. Key enablers were promoting a multidimensional approach by staff and additional support for health professionals to effectively implement the program. Implementation could be promoted by the use of champions, adapting the timing and delivery for different ward cultures, and being cognizant of patient diversity. In addition, staff recommended translating the Safe Recovery Program into different languages to improve accessibility and information uptake to culturally diverse patients. The findings from this study will be used to inform future iterations of the Safe Recovery Program and implementation in hospital settings.
